# Isolation and characterization of novel soil- and plant-associated bacteria with multiple phytohormone-degrading activities using a targeted methodology

**DOI:** 10.1099/acmi.0.000053

**Published:** 2019-08-28

**Authors:** Francisco X. Nascimento, Bernard R. Glick, Márcio J. Rossi

**Affiliations:** ^1^ Departamento de Microbiologia, Laboratório de Bioprocessos, Universidade Federal de Santa Catarina, Florianópolis, SC, Brazil; ^2^ Department of Biology, University of Waterloo, Waterloo, Ontario, Canada

**Keywords:** 1-aminocyclopropane-1-carboxylic acid, salicylic acid, indole-3-acetic acid, plant growth-promoting bacteria, plant–microbe interactions

## Abstract

Ethylene (ET), salicylic acid (SA) and indole-3-acetic acid (IAA) are important phytohormones regulating plant growth and development, as well as plant-microbe interactions. Plant growth-promoting bacteria (PGPB) naturally associate with plants and facilitate plant growth through a variety of mechanisms, including the ability to modulate the concentrations of these phytohormones *in planta*. Importantly, the wide presence of phytohormone degradation mechanisms amongst symbiotic and other soil- and plant-associated bacteria indicates that the ability to modulate phytohormone concentrations plays an important role in bacterial colonization and plant-growth promotion abilities. Obtaining phytohormone-degrading bacteria is therefore key for the development of novel solutions aiming to increase plant growth and protection. In this paper, we report an optimized targeted methodology and the consequent isolation of novel soil- and plant-associated bacteria, including rhizospheric, endophytic and phyllospheric strains, with the ability to degrade the phytohormones, SA and IAA, as well as the ET precursor, 1-aminocyclopropane-1-carboxylic acid (ACC). By using an optimized targeted methodology, we rapidly isolated diverse soil- and plant-associated bacteria presenting phytohormone-degrading abilities from several plants, plant tissues and environments, without the need for prior extensive and laborious isolation and maintenance of large numbers of isolates. The developed methodology facilitates PGPB research, especially in developing countries. Here, we also report, for the first time, the isolation of bacterial strains able to concomitantly catabolize three phytohormones (SA, IAA and ACC). Ultimately, the described targeted methodology and the novel phytohormone-degrading bacteria obtained in this work may be useful tools for future plant-microbe interaction studies, and in the development of new inoculant formulations for agriculture and biotechnology.

## Introduction

Plant growth and development is tightly regulated by internal cues such as phytohormone biosynthesis and signalling [1]. Phytohormones not only regulate plant development and stress responses, but also plant microbiome assembly and general plant-microbe interactions, including beneficial interactions with symbionts (rhizobia) and other plant growth-promoting bacteria (PGPB) that support plant growth under a variety of limiting conditions [2]. In this regard, the phytohormones ethylene (ET), indole-3-acetic acid (IAA) (an auxin) and salicylic acid (SA) are major regulators and central players in plant developmental programmes, the plant microbiome assembly process and general plant-microbe interactions [3–7].

Plant-associated bacteria evolved the ability to modulate the levels of these important hormones (ET, IAA, SA) in soils and plant tissues, and consequently, affect plant growth, development and stress resistance. The bacterial modulation of plant hormone levels can occur in an additive manner, by direct production of phytohormones (e.g. IAA and other auxins), or in a subtractive manner, by the direct catabolism of phytohormones (e.g. IAA and SA) or their precursors (e.g. 1-aminocyclopropane-1-carboxylatic acid, ACC, the ET precursor). While many aspects of bacterial phytohormone production (mainly IAA biosynthesis) have been described and studied in detail [6, 7], much less is understood about the impact of bacterial phytohormone catabolism in beneficial plant-microbe interactions. However, several studies have indicated the important role of bacterial phytohormone catabolism in positively modulating plant growth and symbiotic processes, as well as resistance to biotic and abiotic stresses. For example, many PGPB can decrease both plant ACC and ET levels by producing the enzyme ACC deaminase, which cleaves ACC into ammonia and ⍺-ketobutyrate [8]. PGPB expressing the enzyme ACC deaminase have been shown to promote the growth and nodulation of several plant species under a variety of stress conditions [9, 10]. Some soil- and plant-associated bacteria, such as the *Bradyrhizobium *, *Azoarcus *, *Paraburkholderia *, *Pseudomonas *, *Acinetobacter *, *Arthrobacter* and *Rhodococcus* strains [11–17], can present the ability to catabolize IAA through a variety of biochemical pathways. Moreover, IAA catabolism plays an important role in the plant growth-promoting traits of PGPB such as *Pseudomonas putida* 1290 and *Paraburkolderia phytofirmans* PsJN [12, 16]. The catabolism of SA is a trait found in various soil- and plant-associated bacteria [4, 18, 19] and results from the action of SA hydroxylase and accessory enzymes [18, 20, 21]. The degradation of SA is intimately involved in the colonization abilities of plant-associated bacteria [4].

The presence of ACC, IAA and SA degradation mechanisms amongst symbiotic and plant-associated bacteria predicts a close evolutionary relationship with a plant host and plant growth promotion activities [9]. As such, obtaining ACC-, IAA- and SA-degrading bacteria is key for the study and development of novel solutions aiming to increase plant growth and protection. Nonetheless, obtaining diverse PGPB strains able to catabolize these phytohormones is a difficult task, since using traditional isolation methods usually involves laborious and time-consuming screening of bacteria isolates, which is often unsuccessful.

In order to isolate novel phytohormone-degrading bacteria with agricultural and biotechnological potential, and to overcome previous isolation limitations, we describe and demonstrate an optimized targeted methodology that allows the simple and fast isolation and characterization of soil- and plant-associated bacteria (including rhizospheric, endophytic and phyllospheric strains) with the ability to directly degrade phytohormones (SA and IAA) or their precursors (e.g. ACC, the ET precursor).

## Methods

### Overview of the targeted methodology

**Fig. 1.**
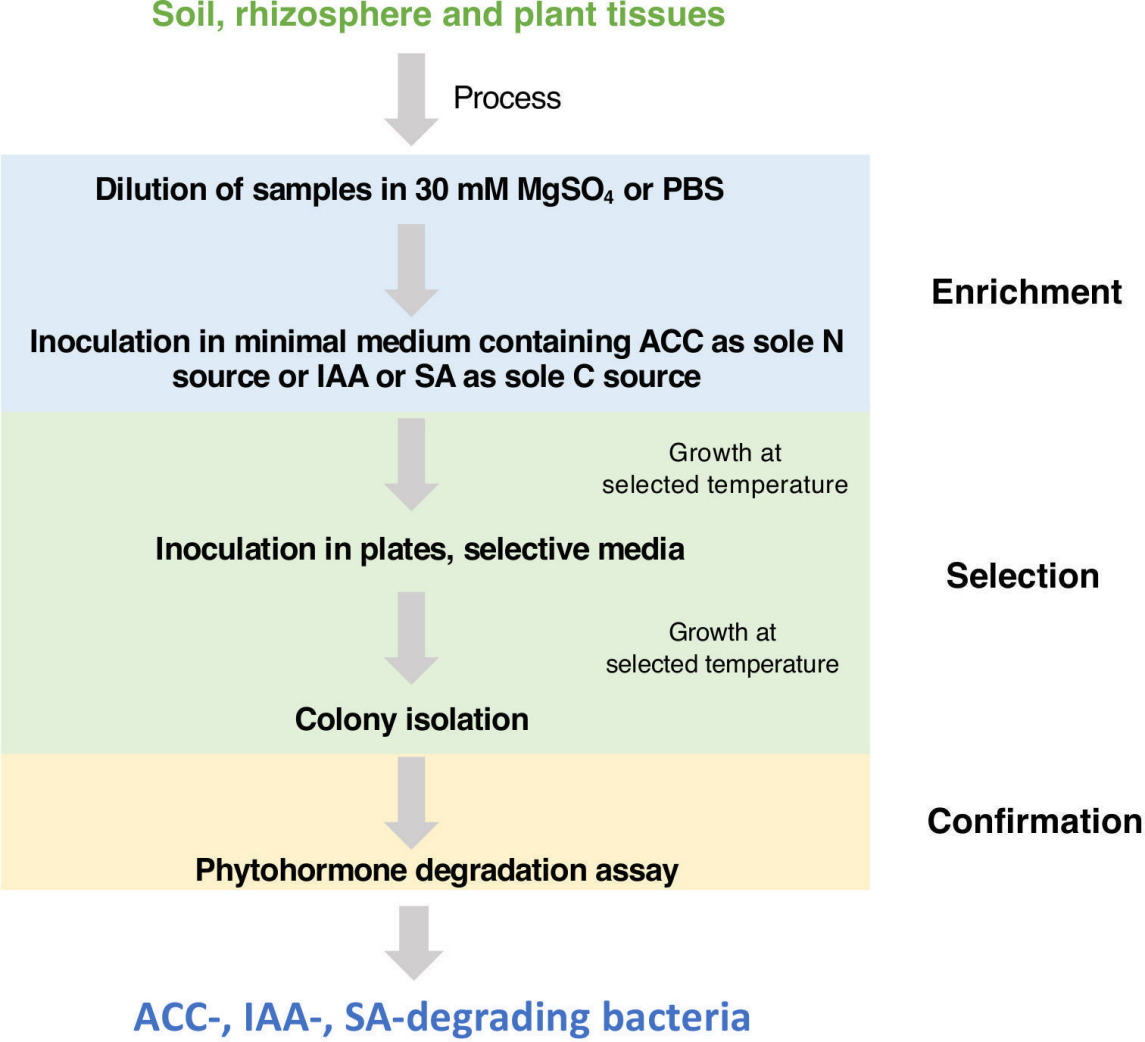
Schematic representation of the developed methodology employed in order to obtain phytohormone-degrading bacteria.

The optimized targeted methodology (Fig. 1) is based on direct bacterial enrichment in minimal medium containing the selected phytohormone as the sole carbon (IAA and SA) or nitrogen (ACC) source, and posterior isolation in selected growth media for the targeting of specific bacterial groups (e.g. *Pseudomonas* Agar F for the isolation of fluorescent *Pseudomonas *, or Yeast Mannitol Agar supplemented with Congo red for the isolation of rhizobia). Phytohormone-degrading abilities are then confirmed based on optimized, simple and affordable methods, using Salkowski’s reagent (IAA determination), Trinder reagent and/or UV fluorescence (SA determination) and an optimized ACC deaminase activity determination protocol. A detailed protocol of the methodology, containing all the necessary steps, reagents and materials, is presented in File S1 in the Supplementary Information file (available in the online version of this article).

### Isolation of phytohormone-degrading bacteria from diverse plant and soil samples

#### Samples and preparation

In order to obtain various bacteria presenting phytohormone-degrading activities, several plant and soil samples obtained from diverse environments and countries were used (Table 1). For this purpose, plants such as *Solanum capsicoides*, *Mimosa scabrella*, *Mimosa bimucronata*, *Mimosa pudica* and *Sesbania virgata*; lower plants, such as an *Sanionia uncinata*, obtained in Antarctica; and soil samples obtained from agricultural and environmental areas (including stress-inducing soils, such as those of mining areas) were exploited (Table 1).

**Table 1.** Bacteria isolated using a targeted methodology and their characterization (identification by 16S rRNA sequencing and analysis, and phytohormone-degrading activities)

**Bacteria ID**	**Accession** **no.#**	**Source**	**Sample collection site**	**Degradation**	ACD activity*
**ACC**	**IAA**	**SA**
* Achromobacter* sp. AB2	MG602707	Antarctic soil	King George Island, Antarctica (62° 16' 48.2" S, 58° 26' 36.5" W)	+	+	+	1.164
* Achromobacter xylosoxidans* SOLR10	MG602708	Solanacea rhizosphere	Florianópolis, Brazil (27° 35' 49.7" S 48° 30' 54.7" W)	+	+	+	1.392
* Arthrobacter* sp. PM3	MG602693	Bermuda grass rhizosphere	Florianópolis, Brazil (27° 26' 55.7"S 48°28'08.3"W)	+	−	−	1.467
* Azorhizobium doebereinerae* SV2	MH925970	*Sesbania virgata* root nodules (interior)	Florianópolis, Brazil (27° 30' 16.4" S 48° 30' 49.7" W)	+	−	−	3.908
* Burkholderia* sp. OPX	MG602709	Unknown tree trunk/fungi-fruit body	Florianópolis, Brazil (27° 35' 57.5" S 48° 31' 03.9" W)	+	−	−	13.125
* Burkholderia* sp. TRE3	MG602704	Acid mine – drainage soil	Criciúma, Brazil (28° 34' 31.0" S 49° 27' 21.8" W)	−	+	+	−
* Lelliottia* sp. AC1	MG602700	*Pinus pinea* leaf	Setúbal, Portugal (38° 29' 06.2" N 8° 56' 38.4" W)	−	+	−	−
* Microbacterium* sp. PM5	MG602705	Bermuda grass rhizosphere	Florianópolis, Brazil (27° 26' 55.7" S 48° 28' 08.3" W)	+	−	−	0.439
* Pantoea* sp. NE1	MG602702	*Sesbania* sp. root nodule (interior)	Florianópolis, Brazil (27° 26' 55.9" S 48° 28' 07.4" W)	+	−	−	5.748
* Pantoea* sp. MSR2	MG602694	*Mimosa scabrella* rhizosphere	Florianópolis, Brazil (27° 26' 55.1" S 48° 28' 07.4" W)	+	−	−	4.793
* Paraburkholderia* sp. MBD1	MH925971	*Mimosa bimucronata* root nodule (interior)	Florianópolis, Brazil (27° 26' 56.7" S 48° 32' 15.8" W)	+	−	−	13.601
* Paraburkholderia* sp. MBS1	MH925972	*M. bimucronata* root nodule (interior)	Florianópolis, Brazil (27° 30' 31.1" S 48° 30' 48.9" W)	+	−	−	4.041
* Paraburkholderia* sp. MP1	MH925968	*Mimosa pudica* root nodule (interior)	Florianópolis, Brazil (27° 30' 16.4" S 48° 30' 49.7" W)	+	−	−	4.179
* Pseudomonas* sp. ACR2	MG602697	Cactaceae rhizosphere	Setúbal, Portugal (38° 29' 02.0" N 8° 58' 09.4" W)	+	−	−	7.679
* Pseudomonas* sp. MS8	MG602701	Shoot of *M. scabrella* (interior)	Florianópolis, Brazil (27° 35' 54.3" S 48° 30' 56.5" W)	+	−	−	3.756
* Pseudomonas* sp. NFX1	MG602706	*Eucalyptus* sp. rhizosphere	Setúbal, Portugal (38° 30' 27.9" N 8°55'44.4"W)	−	+	−	−
* Pseudomonas thivervalensis* PLM3	MG602711	Agricultural soil	Palmela, Portugal (38° 32' 36.9" N 8° 56' 11.0" W)	+	−	−	15.322
* P. thivervalensis* SC5	MG602695	Interior of a *Solanum capsicoides* fruit	Florianópolis, Brazil (27° 34' 22.7" S 48° 25' 21.1" W)	+	−	−	18.592
* Pseudomonas* sp. ACM7	MG602698	Moss rhizosphere	Collins Glacier, Antarctica (62° 09.752' S; 58° 55.825' W)	−	+	−	−
* Pseudomonas* sp. PLM1	MG602710	Agricultural soil	Palmela, Portugal (38° 32' 36.9" N 8° 56' 11.0" W)	+	−	−	10.511
* Pseudomonas* sp. PLMAX	MG602703	Agricultural soil	Palmela, Portugal (38° 32' 36.9" N 8° 56' 11.0" W)	−	+	+	−
* Serratia marcescens* DAMR1	MG602699	Acid mine – drainage soil	Criciúma, Brazil (28° 34' 31.0" S 49° 27' 21.8" W)	−	−	+	−

#16S rRNA GenBank accession number.

*ACC deaminase activity in μmol α-ketobutyrate/mg protein/h.

†+, positive.

‡−, negative.

#### Soil bacteria

Approximately 200 mg of soil was stored in a sterile 50 ml Falcon tube containing a 10 ml solution of 30 mM MgSO_4_ and gently mixed for 30 s using a vortex. The obtained solution was diluted (10^−3^) using sterile 30 mM MgSO_4_ and stored at 4 °C for several days, until further use.

#### Rhizospheric bacteria

The selected plants were removed from soil, the shoot was cut with a sterile scalpel or a similar cutting instrument, and the root stored in a sterile 50 ml Falcon tube using disinfected forceps. A small section of the root (i.e. 5–10 cm) was directly dipped several times in a sterile 10 ml solution of 30 mM MgSO_4_. This solution was diluted (10^−3^) using sterile 30 mM MgSO_4_ and stored at 4 °C for several days, until further use.

#### Root, root nodule, shoot and fruit endophytes

Small sections of plant tissues and root nodules were disinfected by rinsing with 70 % ethanol (1.5 min) and then with 1 % bleach (10 min), followed by five consecutive washes with sterile distilled water. A small section of disinfected plant tissues (2×2 cm), as well as root nodules, was transferred to a sterile microcentrifuge tube containing 1 ml of sterile 30 mM MgSO_4_ and crushed several times with the help of a sterile micropestle. This solution was diluted (10^−3^) using sterile 30 mM MgSO_4_ and stored at 4 °C for several days, until further use.

### Enrichment and isolation of phytohormone-degrading bacteria from diverse plant and soil samples

For the isolation of ACC deaminase-producing bacteria, 50 µl of the solutions obtained in the sample preparation section was inoculated in 5 ml of liquid Dworkin and Foster (DF) or M9 minimal medium (described File S1 in the Supplementary Information file) containing ACC (in a final concentration of 3 mM) as the sole nitrogen source, and incubated at 28 °C in an orbital shaker (150 r.p.m.) for 4–12 days. After observing increased bacterial growth (typically ~5 days), 10 to 20 µl of the bacterial suspension was reinoculated in 5 ml minimal medium containing 3 mM ACC and incubated at 28 °C in an orbital shaker (150 r.p.m.) for 4–12 days. Finally, 10 to 20 µl of the bacterial suspension was plated onto Tryptic Soy Agar, *Pseudomonas* Agar F and *Actinomycetes* Agar (HiMedia), and colonies were isolated.

In order to isolate SA- and IAA-degrading bacteria, 50 µl of the solutions obtained in the sample preparation section was inoculated in 5 ml of liquid DF or M9 minimal medium containing SA or IAA (in a final concentration of 1 mM) as the sole carbon source, and incubated at 28 °C in an orbital shaker (150 r.p.m.) for 4–12 days. The procedure was repeated as described above. After observing increased bacterial growth (typically ~7 days), 10 to 20 µl of the bacterial suspension was plated onto Tryptic Soy Agar, *Pseudomonas* Agar F and *Actinomycetes* Agar (HiMedia), and colonies were isolated.

### Confirmation of bacterial phytohormone-degradation abilities

#### Determination of ACC degradation

Qualitative ACC degradation was confirmed by testing the bacteria isolated (pure cultures) for their ability to grow in minimal medium containing ACC as a sole nitrogen source. Additionally, ACC deaminase activity was tested using a simplified version (described in detail in the Supplementary Information fileFile S1) of the method described by Penrose and Glick [22].

#### Qualitative determination of SA and IAA degradation

Qualitative IAA or SA degradation was confirmed by testing the isolated bacterial cells (pure cultures) for their ability to grow in minimal medium containing IAA or SA as a sole carbon source. Additionally, a SA and IAA degradation test was performed in 24-well plates containing minimal medium supplemented with 1 mM SA or IAA and 0.8 % agar. In this case, 5 µl of an overnight-grown culture (grown in Tryptic Soy Broth medium) was inoculated in the centre of the well. The plate was then incubated for 24 h at 28 °C. SA degradation was identified by examining plates under UV radiation, where wells inoculated with strains unable to degrade SA had a fluorescent appearance, and wells inoculated with strains able to degrade SA did not fluoresce (Supplementary Information fileFile S1). IAA degradation was determined based on the use of the Salkowski’s reagent [23]. The wells containing bacteria unable to degrade IAA changed to a pink colour (negative), while IAA-degrading bacteria removed IAA from the medium and no pink colour developed (positive) (Supplementary Information file File S1).

### Bacterial identification by 16S rRNA sequencing

The 16S rRNA gene sequencing was conducted following genomic DNA extraction from an overnight culture using the GenElute Bacterial Genomic DNA kit (Sigma Aldrich) according to the manufacturer’s instructions. The obtained DNA was sent to the Macrogen company (Republic of Korea), and amplified by PCR using primers 27F (5′-AGAGTTTGATCCTGGCTCAG-3′) and 1492R (5′-GGTTACCTTGTTACGACTT-3′) following the Macrogen PCR amplification and sequencing protocol. The obtained sequences were analysed using Geneious software v 9.0 and submitted to GenBank (the accession numbers can be found in Table 1).

## Results and Discussion

By using a targeted methodology, several ACC-, IAA- and SA-degrading bacterial strains were successfully isolated and characterized in this study (Table 1). These bacteria were obtained from different plants (e.g. *Mimosa*, *Sesbania* and *Solanum*), plant tissues (roots/rhizospheres, root nodules, shoots, leaves and fruits), as well as lower plants (e.g. Antarctic moss) and soils (agricultural, environmental and polluted) obtained from different countries and continents, (including Antarctica), indicating that phytohormone-degrading bacteria are ubiquitous to many environments and are common members of the plant and soil microbiome.

Moreover, the isolated bacteria were identified based on the partial 16S rRNA gene sequence (~1346 bp). Phytohormone-degrading bacteria belonging to different genera (and phyla) were identified, such as *Azorhizobium* (* Alphaproteobacteria *); *Achromobacter *, *Burkholderia* and *Paraburkholderia* (* Betaproteobacteria *); *Lelliottia *, *Pantoea *, *Serratia* and *Pseudomonas* (* Gammaproteobacteria *); and *Arthrobacter* and *Microbacterium* (* Actinobacteria *) (Table 1). This indicated that phytohormone catabolism can be found in a wide range of bacterial genera and species. These results are consistent with the important role of bacterial phytohormone catabolism in general plant-microbe interactions.

Interestingly, several of the isolated bacteria possessed the ability to degrade more than one phytohormone (Table 1). For example, *Achromobacter* sp. strain AB2 isolated from Antarctic soil, and *Achromobacter xylosoxidans* SOLR10, isolated from the rhizosphere of a *Solanaceae* plant in Brazil, possessed the ability to use ACC, SA and IAA as nitrogen and carbon sources, respectively (Table 1). This is the first report of bacteria that are able to degrade three phytohormones. In addition, *Burkholderia* sp. TRE3 (isolated from acid mine drainage soil in Brazil) and *Pseudomonas* sp. PLMAX (isolated from agricultural soil in Portugal) presented the ability to degrade both IAA and SA (Table 1). These results are in agreement with previous reports of IAA and SA degradation by *Pseudomonas* and *Burkholderia* (*sensu lato*), as well as other studies demonstrating the aromatic compound degradation abilities of *Achromobacter *, *Burkholderia* and *Pseudomonas *, and their important role in soil and promoting plant growth [11, 12, 16, 24].

In addition, several rhizobial strains, including both *Alpha* *proteobacteria* (* Azorhizobium *) and *Betaproteobacteria* (* Paraburkholderia *), presenting ACC deaminase activity were isolated (Table 1). ACC deaminase expression increases the nodulation abilities of several rhizobia [25–28] by decreasing ethylene levels, which inhibit the symbiotic nodulation process [3, 29]. Hence, obtaining rhizobia and other free-living bacteria presenting ACC deaminase activity is important for increased nodulation and biological nitrogen fixation in selected leguminous plants. Curiously, *Pantoea* sp. NE1, a root nodule endophyte presenting ACC deaminase activity, was also isolated from a disinfected root nodule of a *Sesbania* plant in Florianópolis, Brazil (Table 1). Recent studies demonstrated that bacterial endophytes presenting ACC deaminase activity increased the nodulation abilities of rhizobia [30, 31], and thus it is likely that *Pantoea* sp. NE1 presenting ACC deaminase activity facilitates the nodulation and nitrogen fixation process.

Some studies have reported the isolation of phytohormone-degrading bacteria (mainly those degrading ACC); however, these were laborious and expensive to conduct. For instance, in an effort to isolate endophytic ACC deaminase-producing bacteria from tomato, Rashid *et al*. [32] isolated 174 bacterial strains, yet only 25 of these (13 %) were able to degrade ACC. Similarly, in a survey in 30 different sites across southern Saskatchewan, Canada, Duan *et al*. [33] found that only 27 out of 233 (11.6 %) isolated rhizobia presented ACC deaminase activity. The fact that only a small portion of the cultivable bacterial community presents ACC deaminase activity makes the isolation of these bacteria a challenging process. On the other hand, the targeted methodology employed in this study allowed the simple, fast and reproducible isolation of various ACC deaminase-producing bacteria from a wide range of plant species, tissues and environments, without the need for extensive and laborious isolation of bacterial isolates. This methodology is based on readily accessible materials that can be used in most microbiology laboratories, and therefore facilitates PGPB research, especially in developing countries.

While the prevalence of ACC deaminase activity has been studied in some plant-associated bacterial communities, not much is understood about the prevalence of IAA- and SA-degrading bacteria or their impact on the microbiome of plants. The methodology developed in this work may also potentiate novel studies on the role of bacterial IAA and SA catabolism in plant growth and stress resistance.

Ultimately, the novel ACC-, IAA- and SA-degrading bacteria obtained in this work can be used in future studies focusing on phytohormone modulation and plant-microbe interactions, as well as for the development of a new generation of inoculants that are suitable for both agricultural and biotechnological applications. New studies regarding the functional and genomic characterization of these new phytohormone-degrading bacteria are being conducted and will unveil the mechanisms behind the phytohormone catabolism and the plant growth promotion abilities of the isolated strains.

## Supplementary Data

Supplementary material 1
