# Supplementary material for: Isolation and characterization of novel soil- and plant-associated bacteria with multiple phytohormone-degrading activities using a targeted methodology
Source: Access Microbiol. 2019 Aug 28;1(7):e000053. doi: 10.1099/acmi.0.000053 (PMC7481731; doi:10.1099/acmi.0.000053)
Supplement: Supplementary material 1 [file acmi-1-053-s001.pdf]

SUPPLEMENTARY INFORMATION

Isolation and characterization of novel soil and plant-associated bacteria with multiple phytohormone-degrading activities using a targeted methodology

Francisco X. Nascimento[1], Bernard R. Glick[2], Márcio J. Rossi[1]

1. Departamento de Microbiologia, Laboratório de Bioprocessos, Universidade Federal de Santa Catarina, Florianópolis SC 88040-900, Brazil
2. Department of Biology, University of Waterloo, Waterloo, ON, Canada N2L 3G1

DETAILED METHODOLOGY FOR THE ISOLATION AND CHARACTERIZATION OF NOVEL SOIL AND PLANT-ASSOCIATED BACTERIA WITH MULTIPLE PHYTOHORMONE-DEGRADING ACTIVITIES

CONSIDERATIONS FOR THE SELECTION OF THE SOURCE MATERIAL

Different source materials can be used for the selection of bacteria colonizing plant tissues, structures (e.g. root nodules) and the rhizosphere (**Table 1**).

Table 1- Plant-associated bacteria and their potential applications

Bacteria	Source	Application examples
Rhizospheric	Soil surrounding roots	General plant-growth promotion and protection, phytoremediation
Root endophyte	Root tissue	General plant-growth promotion and protection, phytoremediation
Root nodule endophyte	Root nodules	Nitrogen fixation, Rhizobia-helper bacteria, indirect increase in nodulation and nitrogen fixation
Shoot endophyte	Shoot tissue	General plant-growth promotion and protection, Nitrogen fixation
Leaf endophyte	Leaf tissue	Aerial tissue growth promotion, plant protection, Nitrogen fixation
Flower endophyte	Flower tissue	Plant reproductive organ development, regulation of flower senescence
Fruit endophyte	Fruit tissue	Regulation of fruit development and senescence, pathogen protection, seed colonization
Seed endophyte	Seeds	Seed germination, seed protection, general plant-growth promotion
Leaf epiphyte	Leaf tissue	Aerial tissue growth promotion, plant protection

Most of the plant-growth promoting bacteria described in the literature are isolated from soil or the rhizosphere of selected plants. However, this can represent a limitation since their consequent application may be limited to soil, plant roots or the external surfaces of plant seeds. Alternatively, endophytic bacteria can be isolated from within plant tissues and this may present several advantages in future applications. Thus for example, plant growth-promoting endophytes can be used to inoculate plants at the flowering stage, which may lead to the bacterial colonization of the newly produced seeds (Mitter et al., 2017). This may not only lead to increased plant growth but also to an increased level of protection against some pathogens, since these endophytes may directly compete with many pathogens that are transmitted via seeds. Bacterial endophytes can also be protected from the competitive soil environment, which may impact bacterial performance (e.g. plant growth promotion

abilities or degradation of xenobiotics) (Hardoim et al., 2015). Therefore, the isolation and selection of endophytes may lead to the development of more efficient inoculants. Leaf-associated bacteria (epiphytes or endophytes) can also be extremely useful, especially at the level of field application. Most of these bacteria can cope with the stresses presented in the leaf environment, which most rhizobacteria cannot endure (e.g. temperature shifts, UV radiation, desiccation) (Vorholt, 2012). Leaf-associated bacteria may also directly compete with many of the plant pathogens colonizing leaf tissues (e.g. *Pseudomonas syringae*, *Xanthomonas* spp.). One of the most important aspects of these bacteria resides in the possibility of their direct application onto leaves (spraying) and their subsequent ability to colonize plant tissues. Spraying is a common agricultural practice (e.g. application of pesticides or herbicides) and may facilitate the acceptance of foliar bacterial inoculants amongst farmers.

ISOLATION OF BACTERIA: NATURAL VERSUS ARTIFICIAL SELECTION SYSTEMS

Plant and rhizosphere samples can be obtained from plants growing in wild and natural habitats (herein termed natural conditions) or by using selected soils, conditions and trap plants (herein termed artificial conditions). Each system has its own advantages and disadvantages and its use may depend on diverse factors, such as, availability of material, reagents, equipment (e.g. growth chambers and greenhouses) and time (**Table 2**).

Table 2- Considerations regarding the isolation of bacteria: natural versus artificial selection systems.

Source	Advantages	Disadvantages
**Natural conditions**	-Representative of field conditions<br>-Increased diversity of plants to sample<br>-Climate and microclimate adaptations<br>-Less expensive<br>-Less time consuming	-Less control of conditions
**Artificial conditions**	-Selection and control of conditions<br>-Selection of plants and specific cultivars<br>-Selection and manipulation of soil conditions<br>-Obtaining bacteria adapted to specific conditions used for commercial applications	-Time consuming<br>-More expensive

Natural conditions are representative of native soils and specific environments (i.e. climate adaptations) and this fact may lead to the easier isolation of bacteria naturally adapted to such situations. Artificial conditions can be created to select for desirable traits. For example, promiscuous leguminous plants are often used as trap plants to isolate rhizobia species

present and adapted to certain soils. Then these strains can be tested for their symbiotic efficiency.

SAMPLE PREPARATION

1. Rhizospheric bacteria

a) (Natural or artificial systems)- Remove the plant from soil, cut the shoot with a scalpel or similar cutting instrument, and store the root in a sealed sterile container (e.g. Falcon tube, sterile plate), using disinfected forceps (ethanol 70%, 2 min, or autoclaved 121°C, 15 min). Store the root and associated soil at 4°C (for up to several days) until further use.

b) (Under sterile conditions) Remove the soil associated with the root system with the help of sterile forceps and store the rhizospheric soil in a sterile 50 ml falcon tube or similar container (e.g. Erlenmeyer flask, plate) containing a sterile solution of 30 mM $MgSO_4$ or 1X Phosphate Buffered Saline (PBS) (the amount may depend on the amount of soil to sample). Usually, only a small amount of soil (100 mg) is necessary to isolate bacteria. However, additional material may be used when working in marginal or degraded soils harboring fewer bacteria.

c) Alternatively, directly dip a small section of the root (i.e. 5-10 cm) several times in a sterile 10 ml solution of 30 mM $MgSO_4$ or PBS.

d) Vortex the solution for 30 secs to break up any soil aggregates.

e) Perform serial dilutions using 30 mM $MgSO_4$ or PBS 1X. Directly use the solutions (described below) or store them at 4°C (for up to several days) for further use.

2. Root and root nodule endophytes

a) Repeat steps a) of the procedure described in 1.

b) Wash the root several times with distilled water to remove soil aggregates and rhizospheric bacteria. Repeat until the root system is free of soil particles.

c) Surface disinfect the root tissue or the root nodule by rinsing it with 70% ethanol and then with 1% bleach. This procedure may vary depending on the plant species and age. For plants with small and thin roots (e.g. tomato) a soft surface disinfection procedure is recommended. This can be accomplished by treating the roots with 70% ethanol for 1.5 min, 1% bleach solution for 10 min and 5 consecutive washes with sterile distilled water. For harder and thicker roots (e.g. tree species) and root nodules, an increased time in the

disinfection solutions is recommended (e.g. 2.5 min in 70% ethanol and 15 min in 1% bleach followed by 5 consecutive washes with sterile distilled water).

d) Crush a small section of the root tissue (i.e. 5-10 cm long) with the help of a sterile mortar and pestle. Add 1 ml of 30 mM $MgSO_4$ or PBS to the crushed tissue. Grind the tissue.

e) Remove the surface disinfected root nodules (3 or 4) from roots with a sterile forceps and transfer to a sterile 2 ml tube containing 500 μl of 30 mM $MgSO_4$ or PBS. Crush the nodules with the help of a sterile micropestle.

f) Perform serial dilutions using 30 mM $MgSO_4$ or PBS 1X. Directly use the solutions (described below) or store them at 4ºC (for up to several days) for further use.

3. Shoot, leaf, flower and fruit endophytes

a) (Natural or artificial systems)- Cut the shoot, leaves, flower (or portions of it) with a sterile scalpel or similar cutting instrument and place it in a sealed sterile container (e.g. Falcon tube, sterile plate), using disinfected forceps. Store the tissues at 4ºC for a short period of time (up to several days).

b) Cut small sections of shoots (i.e. 2 cm long) or leafs (i.e. 2 x 2 cm) with a sterile scalpel.

c) Repeat step c) described in procedure 2.

d) Alternatively, after disinfection, cut small sections (i.e. ~2 cm long) of shoot tissue and place 2 or 3 sections in sterile falcon tubes containing 5 ml of 30 mM $MgSO_4$ or PBS. Incubate overnight at room temperature with shaking (150 rpm). This procedure is useful for the isolation of endophytes from woody tissues that are difficult to grind. Endophytes present in tissues will be released to the liquid medium which can then be used for isolation procedures.

e) Perform serial dilutions using 30 mM $MgSO_4$ or PBS 1X. Directly use the solutions (described below) or store them at 4ºC (for up to several days) for further use.

TARGETED APPROACH: ISOLATION OF PHYTOHORMONE-DEGRADING BACTERIA FROM PLANT AND SOIL SAMPLES

This easy and targeted methodology is based on bacterial enrichment by using a minimal medium containing the selected phytohormone as the sole carbon or nitrogen source. Any of the solutions previously employed in procedures described in the previous section can be used to isolate these bacteria. Using this simple isolation technique, a wide range of phytohormone-degrading bacteria can be easily isolated.

All growth media and stock solutions used in the following section are described at the end of this document.

Enrichment

1. **ACC**

a) Inoculate 20 to 50 μl of the solutions obtained (sample preparation section) in 5 ml of liquid Dworkin and Foster (DF), or M9 minimal medium, containing ACC (in a final concentration of 3 mM) as the sole nitrogen source, and incubate at 28°C (or other selected temperature depending upon the source of the bacterial sample) in an orbital shaker (150 rpm) for 4-12 days. After observing increased bacterial growth (typically ~5 days), 10 to 20 μl of the bacterial suspension can be plated onto specific media and colonies isolated.

2. **SA or IAA**

a) Inoculate 20-50 μl of the solutions obtained (sample preparation section) in 5 ml of liquid DF, or M9 minimal medium, containing SA or IAA (in a final concentration of 1 mM) as the sole carbon source, and incubate at 28°C (or selected temperature) in an orbital shaker (150 rpm) for 4-12 days. After observing increased bacterial growth (typically ~7 days), 10 to 20 μl of the bacterial suspension can be plated onto specific media and colonies isolated.

***Note**: The more diluted the sample the more time that it will take the bacteria to grow in the enrichment medium. On the other hand, more concentrated samples (especially rhizosphere samples) may contain contaminating bacteria that can survive in the enrichment medium even though these bacteria are not able to use phytohormones as sole carbon or nitrogen sources. To decrease the presence of contaminants, each of the enrichment steps can be repeated.*

Isolation

Although a generic growth medium can be used, in an effort to isolate a wide range of phytohormone-degrading bacteria, it is also possible to use specific media and perform a second targeted approach to isolate particular bacterial groups.

Examples:

Pseudomonas: Plate 10 to 20 μl of the enrichment solution in King's B (KB) medium supplemented with 100 μg/ml ampicillin. Most *Pseudomonas* strains produce fluorescent pigments in KB medium and are naturally able to resist high concentrations of ampicillin.

Actinobacteria: Plate 10 to 20 μl of the enrichment solution in Actinomycete isolation agar (AIA). This medium favors the growth of Actinobacteria.

Rhizobia: Plate 10 to 20 μl of the enrichment solution in Yeast mannitol agar (YMA) supplemented with congo red (25mg/L). Most rhizobia present whitish mucoid colonies in this medium.

Note: *Additionally, plates can also be incubated at different temperatures that can promote the growth of specific bacteria (e.g. 7°C for psychrophilic bacteria or 50°C for thermophiles).*

CONFIRMATION OF PHYTOHORMONE DEGRADATION ABILITIES

Determination of ACC degradation

Qualitative ACC degradation can be easily confirmed by testing the bacteria isolated (pure cultures) for its ability to grow in minimal medium containing ACC as sole nitrogen source.

The following steps should be performed in duplicate:

a) Inoculate a colony in 5 ml DF or M9 medium containing 3 mM ACC as the sole nitrogen source (tester).

b) Inoculate a colony in 5 ml DF or M9 medium without any nitrogen source (negative control).

c) Incubate the inoculated media at 28°C and 150-200 rpm for 5 days.

d) Measure and compare the OD_{600} of both bacterial solutions.

e) A positive ACC deaminase activity is found in strains that can grow on minimal medium containing ACC, but not in minimal media without nitrogen source.

Note: *It is preferred to do this experiment in 50 ml falcon tubes or glass test tubs. In plate/well assays (e.g. 96-well plates) the growth medium often evaporates due to the long time needed to perform the experiment.*

It is essential to test the negative control in this experiment. In some instances, some nitrogen-fixing bacteria can grow on minimal medium containing ACC as the sole nitrogen source but can be negative for ACC deaminase activity. In dubious cases a quantitative ACC deaminase activity measurement is necessary. ACC deaminase activity can be tested using a simplified version of the method described by Penrose and Glick (Penrose and Glick, 2003). This can be performed either qualitatively or quantitatively, however, qualitative determination is more accessible for the standard microbiology lab.

Induction of ACC deaminase expression

a) Grow the selected bacteria in 5 ml of a rich medium (e.g. TSB, YMB) in a 50-ml falcon tube until luxuriant growth is achieved. This depends on the bacterial strain. Usually,

Pseudomonas strains grow very well in 24 h, but other strains, such as, rhizobia or some actinobacteria grow more slowly (48 to 72 h). Incubate at 28ºC, 150-200 rpm.

b) Centrifuge the 50-ml falcon tube at 4000 rpm in a benchtop centrifuge for 10 min and discard the supernatant.

c) Suspend and wash the cell pellet in either 5 ml DF or M9 minimal medium without a nitrogen source. Centrifuge at 4000 rpm for 10 min. Discard the supernatant.

d) Suspend the cell pellet in either 5 ml DF or M9 minimal medium containing 3 mM ACC as the sole nitrogen source. Incubate for 24 or 48 h at 28ºC, 150-200 rpm. This step induces ACC deaminase activity. Be consistent with the incubation time.

e) Centrifuge the tube at 4000 rpm for 10 min and discard the supernatant.

f) Suspend the cell pellet in 1 ml 0.1 M Tris-HCl pH 8.0 and transfer it to a 1.5 ml tube. Centrifuge the suspended cells at 10000 rpm for 1.5 min in a micro-centrifuge.

g) Remove the supernatant and suspend the cells in 400 μl of 0.1M Tris-HCl pH 8.0.

ACC deaminase activity determination

a) Add 20 μl toluene and vortex for 30 seconds (cell permeabilization). This step is crucial for effectively measuring ACC deaminase activity. Some bacterial strains are more resistant to the procedure; in that case small glass beads can be added to the lysate (1:10 v/v) to help disrupting the bacterial cell wall and membrane. Vortex for additional 30 seconds. Note that ACC deaminase is a cytoplasmic enzyme (Jacobson et al., 1994).

b) Dispense 50 μl of lysate into 1.5 ml centrifuge tubes: Two tubes for lysate + ACC (tester); Two tubes for lysate and no ACC (negative control). Also include an internal control: one tube containing 50 μl 0.1M Tris-HCl pH 8.0 + ACC. Save the rest of the lysate at 4ºC (for up to a few days) or -20ºC (for longer periods) for protein concentration measurements or assay repetition.

c) Add 5 μl of 0.3M ACC to each 1.5 ml tube containing 50 μl of lysate (except for the negative controls of each sample) and vortex, approximately 5 secs.

d) Incubate at 30°C for 30 min.

e) Add 500 μl 0.56M HCl and vortex, ~5 sec, and centrifuge for 5 min at 10000 rpm.

f) Prepare standards 0.05-0.5 μmol α-ketobutyrate dissolved in 0.1M Tris-HCl pH 8.0 buffer.

g) Add 500 μl supernatant or standard to a glass test tube (13 x 100 mm) and then add 400 μl 0.56M HCl.

Derivation and quantification of α-ketobutyrate

a) Add 150 μl DNP reagent (0.2% 2,4-dinitrophenylhydrazine in 2 N HCl) and vortex for ~5 sec

b) Incubate at 30°C for 30 min.

c) Add 1 ml 2 N NaOH and vortex for ~5 sec

d) Decant into cuvettes and read the OD at 540 nm. Use 50 μl Tris HCl + 900 μl 0.56 M HCl + 150 μl DNP + 1 ml 2 N NaOH as a blank.

Note*: The derivatization step does not account for ACC deaminase activity. In this step, the enzyme is inactive due to the acidic pH, and the unique purpose is to derivatize phenylhydrazine to phenylhydrazone.*

Protein content measurement

Measure protein content of 50 μl lysate. This can be achieved by using the Bradford reagent following the manufacturers specification and using a Bovine Serum Albumin (BSA) standard curve.

Final representation of ACC deaminase activity

The final ACC deaminase activity should be expressed in μmol α-ketobutyrate/mg protein/hour. It is calculated in the following manner:

α-ketobutyrate in sample = [OD_{540} sample (sample + ACC)] – [OD_{540} negative control (sample without ACC)]. Use the α-ketobutyrate standard curve to calculate the correct α-ketobutyrate value.

The obtained α-ketobutyrate value is divided by the amount of protein present in the 50 μl lysate. This value is then multiplied by 2 since the assay for ACC deaminase activity was determined in only half an hour.

Alternatively, a qualitative estimation of ACC deaminase activity can be made by observing the production of α-ketobutyrate (**Figure 1**). This does not require either a standard curve or protein quantification.

Relative α-ketobutyrate in sample = [OD_{540} sample (sample + ACC)] – [OD_{540} negative control (sample without ACC)].

If relative α-ketobutyrate in a sample is > 0 then the sample is positive for ACC deaminase. Nevertheless, these values need to be interpreted carefully. Most times values close to 0

(ranging from 0 to 0.08) are deemed to represent non-specific enzymatic activities from ACC deaminase related enzymes. This occurs frequently in Enterobacteriaceae, and for example, in some *Pseudomonas* and *Bacillus* spp. which e.g. possess D-cysteine desulfhydrase.

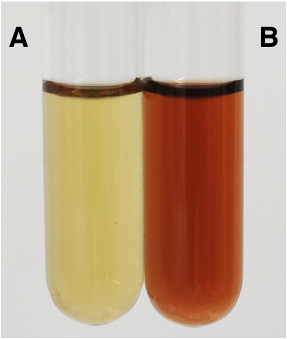

Figure 1- Representation of an ACC deaminase activity measurement (qualitative). **A)** no ACC deaminase activity (no alpha-ketobutyrate present in the sample); **B)** high ACC deaminase activity (high concentrations of alpha-ketobutyrate present in the sample).

Qualitative determination of SA and IAA degradation

Qualitative IAA or SA degradation can be easily confirmed by testing the isolated bacterial cells (pure cultures) for their ability to grow in minimal medium containing IAA or SA as a sole carbon source.

The following is typically performed in duplicate:

a) Inoculate a small amount of a bacterial colony in 5 ml DF or M9 medium containing 1 mM IAA/SA as the sole carbon source (tester).

b) Inoculate a small amount of a bacterial colony in 5 ml DF or M9 medium without any carbon source (negative control).

c) Incubate the bacterial cell suspension at 28°C, 150 rpm for 5 days (or more depending on the bacterium).

d) Measure and compare the OD_{600} of both bacterial solutions, i.e. **a** and **b** above.

A positive SA/IAA degradation activity is inferred from strains that can grow on minimal medium containing SA/IAA, but not in minimal media without an added carbon source.

SA degradation test

Alternatively, the SA degradation test can be performed in 48 or 24-well plates containing minimal medium supplemented with 1 mM SA and 0.8% agar (e.g. 24-well plates = 1 ml M9

medium containing 1 mM SA as sole carbon source per well). In this case, 5 µl of an overnight grown culture (grown in general rich medium) is inoculated in the center of the plate/well. The plate is then incubated for 24-48 h at 28ºC.

SA is fluorescent under UV radiation, so, SA degradation can easily be identified by examining plates under UV radiation. A UV transilluminator, commonly used in molecular biology procedures, may be employed for this purpose. The wells inoculated with strains unable to degrade SA appear fluorescent (**Figure 2A**) while the wells containing strains that can degrade SA will not fluoresce (**Figure 2B**).

Alternatively, the Trinder reagent (described in the supplementary information) can be added to the medium (1 ml Trinder reagent/per well of a 24-well plate) and then incubated for 20 to 30 min. The Trinder reagent is commonly used for the detection of SA (Trinder, 1954). If SA is present, the medium will change color (from yellow to purple) (**Figure 2C**). Bacteria that can degrade SA remove it from the medium and hence no color development is observed (**Figure 2D**).

Note: *Given the tendency of the Trinder reagent-SA complex to fade, the Trinder plate must be examined immediately after the incubation process.*

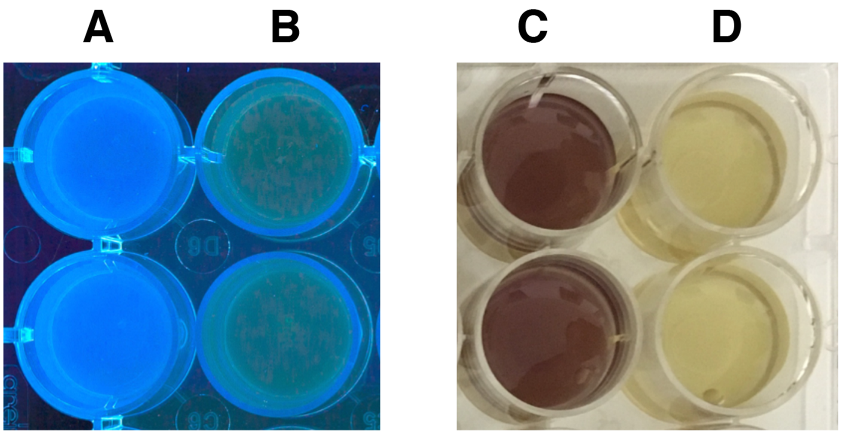

Figure 2- Determination of bacterial SA-degrading abilities in 24-well plates containing solid (0.8% agar) M9 minimal medium and 1 mM SA as sole carbon source, inoculated with 5 µl of a bacterial solution, and incubated for 24 h at 28ºC. **A)** Negative (fluorescence under UV, no degradation of SA); **B)** Positive (no fluorescence under UV, degradation of SA).; **C)** Negative (purple colour indicates the complexation of the Trinder reagent and SA, no degradation of SA); **D)** Positive (no purple colour indicates the absence of salicylate and no complexation of the Trinder reagent, degradation of SA occurred).

IAA degradation test

An IAA degradation test can be performed in 48 or 24-well plates containing minimal medium supplemented with 1 mM IAA and 0.8% agar (e.g. 24-well plates = 1 ml M9 medium containing 1 mM IAA as sole carbon source per well). In duplicate, add 5 μl of an overnight culture (grown in general rich medium) in the center of the well. The plate is then incubated for 24-48 h at 28°C.

The detection methodology is based on the use of the Salkowski reagent that is widely used in the determination of IAA production in bacterial culture medium (Glickmann and Dessaux, 1995). After incubation and growth, a solution of Salkowski reagent (described below) can be added to the solid medium (1 ml Salkowski reagent/per well of a 24-well plate) and further incubated for 1 hour at room temperature. Plates/wells containing bacteria unable to degrade IAA will change to a pink color (negative) (**Figure 3A**). Bacteria able to degrade IAA will consume all of the available IAA and no color development will be observed (positive) (**Figure 3B**).

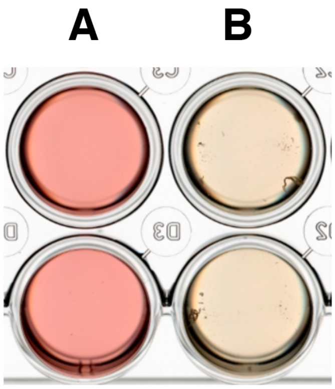

Figure 3- Determination of bacterial IAA-degrading abilities in 24-well plates containing solid (0.8% agar) M9 minimal medium and 1 mM IAA as sole carbon source, inoculated with 5 μl of a bacterial solution, and incubated for 24 h at 28°C. **A)** Negative (pink colour indicates the complexation of the Salkowski reagent and IAA, no degradation of IAA); **B)** Positive (the absence of colour indicates the absence of IAA and no complexation of the Salkowski reagent, degradation of IAA occurred).

REFERENCES

Glickmann, E., Dessaux, Y., 1995. A critical examination of the specificity of the salkowski reagent for indolic compounds produced by phytopathogenic bacteria. Appl. Environ. Microbiol. 61, 793–6.

Hardoim, P.R., Overbeek, L.S. van, Berg, G., Pirttilä, A.M., Compant, S., Campisano, A., Döring, M., Sessitsch, A., 2015. The Hidden World within Plants: Ecological and Evolutionary Considerations for Defining Functioning of Microbial Endophytes. Microbiol. Mol. Biol. Rev. 79, 293–320. https://doi.org/10.1128/MMBR.00050-14

Jacobson, C.B., Pasternak, J.J., Glick, B.R., 1994. Partial purification and characterization of 1-aminocyclopropane-1-carboxylate deaminase from the plant growth promoting rhizobacterium *Pseudomonas putida* GR12-2. Can. J. Microbiol. 40, 1019–1025. https://doi.org/10.1139/m94-162

Mitter, B., Pfaffenbichler, N., Flavell, R., Compant, S., Antonielli, L., Petric, A., Berninger, T., Naveed, M., Sheibani-Tezerji, R., von Maltzahn, G., Sessitsch, A., 2017. A new approach to modify plant microbiomes and traits by introducing beneficial bacteria at flowering into progeny seeds. Front. Microbiol. 8, 11. https://doi.org/10.3389/fmicb.2017.00011

Penrose, D.M., Glick, B.R., 2003. Methods for isolating and characterizing ACC deaminase-containing plant growth-promoting rhizobacteria. Physiol. Plant. 118, 10–15. https://doi.org/10.1034/j.1399-3054.2003.00086.x

Trinder, P., 1954. Rapid determination of salicylate in biological fluids. Biochem. J. 57, 301–3.

Vorholt, J. a, 2012. Microbial life in the phyllosphere. Nat. Rev. Microbiol. 10, 828–40. https://doi.org/10.1038/nrmicro2910

MEDIA AND REAGENTS

Stock ACC

Stock aliquots of ACC (0.3M) can be prepared by diluting ACC in water and then filter sterilizing the diluted ACC before storing the aliquots at -20ºC (for short term use) or -80ºC (for long term storage).

Stock SA and IAA

Stock aliquots of SA (0.1M) and IAA (0.1M) can be prepared by diluting SA or IAA in a 4:1 (v:v) solution of water and 1N NaOH and then filter sterilizing the diluted phytohormones and storing them at -20ºC.

Media, grams per liter:

Dwarkin and Foster (DF) minimal medium

Base:

KH_2PO_4 4.0 g
Na_2HPO_4 6.0 g
$MgSO_4 .7H_2O$ 0.2g
Glucose 2.0 g
Gluconic acid 2.0 g
Citric acid 2.0 g
$(NH_4)2SO_4$ (nitrogen source) 2.0 g (remove if testing ACC as nitrogen source).

Trace elements solution:

In 100 ml sterile distilled water dissolve:
H_3BO_3 10 mg
$MnSO_4.H_2O$ 11.19 mg
$ZnSO_4.7H_2O$ 124.6 mg
$CuSO_4.5H_2O$ 78.22mg
MoO_3 10mg

Iron solution:

dissolve 100 mg of $FeSO_4$.7H2O in 10 ml sterile distilled water

Final Media:

Add 0.1 ml of each of the solutions of trace elements and iron to the base medium, to a final volume of 1 liter.
Adjust pH to 7 with KOH.

M9 minimal medium

Na_2HPO_4 6.0 g
KH_2PO_4 3.0 g
NaCl 0.5 g
NH_4Cl 1g
pH 7.4

Tryptic Soy Agar (TSA)

Tryptone 17 g
Soy Peptone 3 g

NaCl 5 g
K_2HPO_4 2.5 g
Glucose 2.5 g
Agar 15 g

Yeast mannitol agar (YMA)
Yeast extract 1g
Mannitol 10 g
K_2HPO_4 0.5 g
$MgSO_4$ 0.2 g
NaCl 0.1
Agar 15 g

Actinomycete isolation agar (AIA)
Sodium caseinate 2.0 g
L-Asparagine 0.1 g
Sodium propionate 4.0 g
K_2HPO_4 0.5 g
$MgSO_4$ 0.1 g
$FeSO_4$ 0.001 g
Agar 15 g

Pseudomonas agar (King's B)
Casein hydrolysate 10.0 g
Proteose peptone 10.0 g
K_2HPO_4 1.5 g
$MgSO_4$ 1.5 g
Agar 15.0 g
Glycerol 10 ml

Trinder's Color Reagent.
Dissolve 40g of mercuric chloride in 850 mL of water by heating. Cool the solution and add 120 mL of 1N HCL and 40g of ferric nitrate. When all the ferric nitrate has dissolved, dilute the solution to 1L with water.

Salkowski's Reagent
4.5 g/L of $FeCl_3$ in 10.8 M H_2SO_4.

Your stock solution of H_2SO_4 is calculated to be 17.822 M based on a density of 1.84 g/mL, a formula weight of 98.08 g/mol, and a concentration of 95% w/w.

To make a 10.8 M solution, slowly add 605.986 mL of your H_2SO_4 stock solution to 250 mL deionized water. Adjust the final volume of solution to 1000 mL with deionized water.
